# Examining Associations between Source of Cancer Information and Mammography Behavior among Black Church-Going Women

**DOI:** 10.3390/ijerph192013004

**Published:** 2022-10-11

**Authors:** Brian J. Carter, Tzuan A. Chen, Dalnim Cho, Shahnjayla K. Connors, Ammar D. Siddiqi, Lorna H. McNeill, Lorraine R. Reitzel

**Affiliations:** 1 Department of Health Disparities Research, The University of Texas MD Anderson Cancer Center, Houston, TX 77030, USA; 2 Department of Psychological, Health, and Learning Sciences, University of Houston, 491 Farish Hall, Houston, TX 77204, USA; 3 HEALTH Research Institute, University of Houston, 4349 Martin Luther King Boulevard, Houston, TX 77204, USA; 4 Department of Social Sciences, University of Houston-Downtown, Houston, TX 77002, USA; 5 Department of Biosciences, Rice University, 6100 Main St., Houston, TX 77005, USA

**Keywords:** cancer information, information source, mammogram, breast cancer, cancer screening, cancer health equity, church-goers, racial health disparities

## Abstract

Black women have a slightly lower breast cancer incidence rate than White women, but breast cancer mortality is approximately 40% higher among Black women than among White women. Early detection by mammography may improve survival outcomes. Outlets providing information on cancer and cancer screening often present data, including mammography recommendations, that are unreliable, accessible, and/or inconsistent. We examined associations between sources of cancer information and mammography behavior among Black church-going women. A logistic regression model was used to examine associations between self-reported preferred source of cancer information (provider, cancer organization, social network, internet, or other media (e.g., books, magazines)) and self-reported most recent source of cancer information (same categories as preferred sources), respectively, and having received a mammogram within the prior 12 months. Participants were 832 Black women over 40 years old, recruited from three churches in Houston, Texas. Data were collected in 2012. Overall, 55.41% of participants indicated their preferred source of cancer information was a provider, 21.88% the internet, 11.54% other media, 10.22% a cancer organization, and 0.96% their social network. In contrast, 17.88% of participants indicated their most recent source of cancer information was a provider, 63.02% the internet, 12.04% other media, 4.50% a cancer organization, and 2.55% their social network. About 70% of participants indicated receiving a mammogram in the prior 12 months. Results indicated that women who most recently sought information from the internet had lower odds of having a mammogram than those who most recently sought information from a provider (aOR: 0.546, CI_95%_: 0.336–0.886, *p* = 0.014). These results reveal an opportunity to advance health equity by encouraging Black church-going women to obtain cancer information from providers rather than from the internet as a method to enhance mammography use. These results also reveal an opportunity to investigate what modifiable social determinants or other factors prevent Black church-going women from seeking cancer information from their preferred source, which was a provider for the majority of the sample, and designing interventions to better actualize this preference.

## 1. Introduction

### 1.1. Breast Cancer Disproportionately Kills Black Women

Breast cancer is the second leading cause of cancer death among women in the United States (U.S.), trailing only lung cancer [1]. One in every eight American women will develop invasive breast cancer over the course of her lifetime [1]. The American Cancer Society estimates that 51,400 women will be diagnosed with new cases of ductal carcinoma in situ in 2022, while 287,850 will be diagnosed with more invasive breast cancers [1]. The disease will claim the lives of 43,250 women during the year [1]. Those women will be disproportionately Black.

Black women have a 40% higher breast cancer mortality rate than White women (28.2 per 100,000 compared to 20.1 per 100,000) despite Black women having a slightly lower breast cancer incidence rate (129.8 per 100,000 compared to 133.8 per 100,000) [2]. In fact, the breast cancer mortality rate among Black women is higher than that among women from any other racial/ethnic group [2]. This is at least in part because Black women with breast cancer are less likely to be diagnosed with early stage breast cancer than White women [3]. Rather, Black women diagnosed with breast cancer are more likely than White women to present with tumors that have metastasized and are larger than 2 cm in diameter [4]. Tumors found in Black women are also more likely to present with markers of biological aggressiveness (i.e., triple-negative cancers, lymph node metastases, and distant metastases) than tumors found in White women [3].

Improved mammogram usage may enable Black women to have breast cancers detected at earlier, less malignant stages [5]. Stage at diagnosis, however, does not account for all of the disparity in breast cancer deaths. Black women diagnosed with stage I breast cancer still have a lower 7-year survival rate than White women diagnosed with the same [3]—but early detection represents an opportunity to prolong the lives of many women of all racial/ethnic backgrounds. Early stage cancers are easier to treat and less fatal [6]. Black women diagnosed with localized breast cancers have a 96.2% 5-year relative survival rate, whereas those diagnosed with regional or distant breast cancers have 5-year relative survival rates of 77.9% and 20.5%, respectively [7]. According to American Cancer Society data compiled in 2018, 74% of Black women ages 50-74 had had a mammogram at any point within the prior two years [8]. That rate fell short of the Healthy People 2020 target of 81.1% [9] and falls short of the Healthy People 2030 target of 80.5% [10]. 

### 1.2. Breast Cancer Screening Guidelines Abound

The U.S. Department of Health and Human Services opted to set its Healthy People 2020 and 2030 breast cancer screening targets, for women at average risk of breast cancer, based on females aged 50 to 74 receiving a mammogram within the past two years [9,10]. However, screening guidelines for such women vary depending on the entity promulgating them.

The U.S. Preventive Services Task Force (USPSTF) recommends that women ages 40–49 decide for themselves whether to have mammograms and advises women ages 50–74 to have biennial mammograms [11]. The American Cancer Society recommends that women ages 40–44 decide for themselves whether to have mammograms, women ages 45–54 have annual mammograms, and women ages 55 and older to transition to biennial mammograms [12]. The American College of Obstetricians and Gynecologists recommends annual or biennial mammograms for women ages 40 and older [13]. 

The American College of Radiology, the National Comprehensive Cancer Network, and the Society of Breast Imaging each recommend annual mammograms for all women ages 40 and older [14,15]. The University of Texas MD Anderson Cancer Center (MDACC) and Memorial Sloan Kettering Cancer Center (MSKCC) are both National Cancer Institute-Designated Cancer Centers [16]. They are ranked the top two hospitals in America for cancer care by U.S. News & World Report [17] and the top two in the entire world by Newsweek [18]. Both MDACC and MSKCC recommend annual mammograms for all women ages 40 and older [19,20].

### 1.3. Growing Support for Annual Mammograms for Black Women Beginning at Age 40

Black women are disadvantaged by any recommendations to start mammography at age 50 [21]. Approximately 23% of breast cancer cases in Black women are diagnosed at younger than that age, compared to 16% for White women [22]. Though there is variation in mammography guidelines, the majority of the prominent outlets recommend annual mammograms for women ages 40 and older [13,14,15,19,20]. This is consistent with the prevailing practice in clinical medicine, where most physicians recommend mammograms for woman at average risk ages 40 and older [23]. Notably, none of the promulgated guidelines tailor their screening recommendations based on a woman’s race/ethnicity [24,25] despite known disparities in stage at diagnosis [3,4] and mortality for Black women with breast cancers [2]. This is, unfortunately, consistent with the research underpinning those recommendations; research in which Black women are underrepresented [22].

One recent study led by a team of researchers from the University of Michigan Medical School suggested that Black women in particular can benefit from initiating breast cancer screening at age 40. The study used an established model from the Cancer Intervention and Surveillance Modeling Network [26] and found that beginning biennial mammography for Black women at age 40 compared to age 50 for White women would reduce the breast cancer mortality disparity by 57% [27].

Another recent study conducted by diagnostic radiologists from Michigan’s largest hospital system agreed that Black women should begin mammography by age 40, noting that beginning at age 50 is disadvantageous to Black women [21]. The authors of that study, citing the prevalence of more aggressive cancer subtypes among Black women, concluded that Black women ages 40 and older should have annual mammograms [21]. They recommended that all organizations promulgating mammography guidelines strongly consider promoting 40 as the age of initiation [21].

### 1.4. The Trouble with Online Information Generally

The proliferation of information during the 21st century has created an opportunity for patients to change how they interact with the healthcare system. Greater access to cancer screening and other health information ostensibly enables patients to educate themselves and make well-informed decisions about their care. However, the information available may be unreliable, inconsistent, and/or inaccessible. That can be true of information from any source, but the internet, being the most frequently used source of health information among U.S. adults [28], is of particular concern. 

Patients overwhelmingly report health care providers as their most trusted source of cancer information and in-person meetings with a health professional as their preferred way to receive cancer information [29]. Yet, the majority turned to the internet when they most recently wanted cancer information [29]. Patients have articulated several reasons for this behavior. First, the monetary cost of searching the internet is much lower than that of seeing a physician [30]. Second, the internet is always available [31]. Patients can seek immediate answers to questions keeping them awake at night rather than waiting several days or weeks for a doctor’s appointment. Third, the internet offers privacy, sparing patients the anxiety that may accompany discussing personal health issues with others [31]. Fourth, the internet allows patients to modulate the type and level of information they receive at any given time [31]. Some patients may want a bulleted list of symptoms rather than an in-depth analysis of a disease (and vice versa). Others may want to avoid information that scares them. Lastly, patients feel almost obligated to seek information from the internet, either to lessen the information asymmetry between them and their doctors or because they feel it would be irresponsible not to consume as much information as possible about their health [31].

The explanations described above illustrate the utility of internet health information and why patient reliance on such information is likely here to stay. They do not, however, save internet health information from its many shortcomings. Most of those shortcomings can be categorized as either impugning the reliability of such information or limiting its accessibility. Factors impugning reliability include that most such websites do not provide references or otherwise disclose the source of their data, nor do they disclose their data’s timeliness [32]. Further, websites are generally not peer-reviewed [33]. Factors limiting accessibility include the cultural sensitivity and reading level employed in producing health information websites. Studies have found that cancer information websites tend to display only limited cultural sensitivity, neglecting to address racial- or ethnic-specific perceptions of cancer risk or cultural beliefs about health [34]. One study noted that the American Cancer Society’s website was culturally sensitive but contained many pages that were difficult to access for people with low literacy [35]. Cancer information websites tend to require at least a 10th-grade reading level, with more than half requiring college-level reading [36]. The average reading level of cancer websites is grade 13.7 [37]. The high reading level of cancer information websites may present a challenge to many Black patients, with 67% of Black adults having basic or below basic literacy skills according to the most recent National Assessment of Adult Literacy [38].

### 1.5. Online Recommendations Foster Confusion and Mistrust among Black Women

There is evidence that health information obtained online has a significant influence on patients’ behaviors, including whether they make an appointment to see a doctor [39]. This influence, in light of the abundance of (potentially conflicting) breast cancer screening guidelines [11,12,13,14,15,19,20], increased reliance on the internet to find those guidelines [28,29], and the unreliableness and inaccessibility of online cancer screening information [32,33,34,35,36,37,38], may be pernicious to Black women.

A study led by a team from MD Anderson Cancer Center and published earlier this year found that the abundance of different screening recommendations causes racially/ethnically diverse women to question the motives of those making the recommendations [40]. Further, Black participants noted concerns about their race/ethnicity not being represented in the data informing the recommendations [40]. The latter is especially troublesome given that none of the relevant agencies tailor their screening recommendations based on a woman’s race/ethnicity [24,25].

The breast cancer screening information infrastructure is thus one in which Black women are likely to look to the internet for such information and be confronted with a plethora of websites from organizations that they are less likely than White women to trust [11,12,13,14,15,19,20,28,29,40]. If/when they do choose a website from those available, they will find it untailored to their race and potentially unreliable [24,25,32,33]. They are more likely than White women to find that websites lack sensitivity toward their culture and/or are written above their reading level [34,35,36,37,38]. Despite those shortcomings, information gleaned is then likely to significantly influence their behavior [39]. This disconcerting reality underscores the importance of research into associations between cancer information sources and mammography behavior.

### 1.6. The Present Paper

The current study was designed to examine associations between sources of cancer information and mammography behavior among Black church-going women. The Black Church has long been a pillar of the Black community. It has a storied history of involvement in community health initiatives [41,42,43] and is thus well-positioned to lead or assist in implementing any resulting recommendations. Among Black women in America, 84% say that religion is very important or somewhat important in their lives [44], with 65% attending church services at least a few times a year [45]. Many studies have investigated associations between preferred sources of cancer information and factors not including mammography behavior among Black woman [46,47,48,49,50,51]. Another examined associations between mammography behavior and other factors among Black church-going women (not including source of cancer information) [52]. Few have investigated the association between source of cancer information and mammography behavior among these women. A study similar to the current study was published in 1998 [53], the year that Google went live and only 41% of American adults accessed the internet [54,55]. Internet usage has changed drastically in the past 24 years. That study also did not focus on Black or church-going women [53]. 

The current study investigates associations between preferred source of cancer information and most recent source of cancer information, and mammography behavior among Black church-going women in an era when 93% of American adults use the internet and Google processes more than 3.5 billion searches per day [56,57]. It tests two hypotheses: (1) Black church-going women who prefer to receive cancer information from the internet are less likely to have a mammogram in the past year than women who prefer to receive cancer information from a doctor or healthcare provider and (2) Black church-going women who most recently sought cancer information from the internet are less likely to have had a mammogram in the past year compared to women who most recently sought cancer information from a doctor or healthcare provider. Understanding more about these associations may help those designing interventions to prompt earlier and/or more frequent mammography utilization, and thus early breast cancer detection, among Black church-going women.

## 2. Materials and Methods

### 2.1. Study Design

The underlying parent study, called Creating a Higher Understanding of cancer Research and Community Health (Project CHURCH), sought to engage Black adults as partners in the research process to better understand disparities in cancer prevention risk factors. Project CHURCH started in 2008 with a single large church in Houston, Texas with a predominantly Black congregation. Study procedures were informed by a church and scientific advisory board comprised of three faculty members from MDACC’s Department of Health Disparities Research and eight church members selected by the initial Church’s pastor. The project expanded in 2012, adding two smaller churches primarily to broaden the socioeconomic diversity of the sample. Those two churches were also located in Houston and predominantly Black. Each church agreed to participate in data collection designed to better understand cancer risk factors among Black adults. Participants were recruited via flyers posted on the church websites, in the church newsletters, at the churches, and in video announcements aired during church services. Participants were also recruited via in-person solicitation at events such as choir practice, church health fairs, and bible study. To participate in the study, individuals had to self-identify as Black or African American (hereafter, Black), be at least 18 years old, have a working telephone number and address, and attend one of the three churches in the study. Participants completed computer-assisted surveys in person at their church and were given USD 30 debit cards for their efforts. A previous publication describes the design of the parent study, including a discussion of the participating churches and their congregants, in greater detail [41].

For the current data analysis, data from the year 2012 were used, which meant that the 4th year of data was used from the original church participating in the study and the 1st year of data was used for the other two, later onboarding churches. Only women ages 40 and older were included in the analytic sample.

This study was approved by the IRBs associated with MDACC (protocol code 2007-0970, approved 2/28/08; protocol code 2012-0051, approved 2/14/12) and the University of Houston (protocol code 14423-EX, approved 7/10/14).

### 2.2. Measures

#### 2.2.1. Preferred Source of Cancer Information

Participants self-reported from whom or what single source they preferred to receive cancer information. Options included a doctor or health care provider, a cancer organization (incl. 1-800 numbers), their social network (i.e., friends/family or other church members), the internet, or other media (i.e., books, brochures, pamphlets, magazines, newspapers, and the library).

#### 2.2.2. Most Recent Source of Cancer Information

Participants self-reported where they went first the most recent time they wanted cancer information. Options included a doctor or health care provider, a cancer organization (incl. 1–800 numbers), their social network (i.e., friends/family or other church members), the internet, or other media (i.e., books, brochures, pamphlets, magazines, newspapers, and the library).

#### 2.2.3. Mammography Behavior

Mammography behavior was measured by asking participants whether they had ever had a mammogram and, for those selecting “yes,” when they had their most recent mammogram. Those reporting having received a mammogram one year ago or less were deemed to have engaged in mammography behavior recommended by the majority of promulgating entities [13,14,15,19,20], as discussed above.

#### 2.2.4. Covariates

Participants self-reported age (entered into a blank form), education (≤high school, some college, ≥Bachelor’s degree), marital status (married or living with a partner; all others), continuous health insurance coverage for the prior 12 months (yes, or no), annual household income (<USD 40,000, USD 40,000–USD 79,999, ≥USD 80,000), employment status (employed, unemployed), and personal and/or family history of cancer, respectively (yes or no). Participants also indicated (1) how likely they thought it was that they would develop cancer in the future (cancer likelihood; very low, somewhat low, moderate, somewhat high, very high), (2) their likelihood of getting cancer compared to the average person (cancer relative risk; more likely, less likely, about as likely), and (3) how often they worried about getting some type of cancer (cancer worries; never, rarely, sometimes, often, all the time).

Participants self-reported perceived social support. Social support was measured via the International Support Evaluation List (ISEL), a 12-item list that measures the perceived social support across three subscales: appraisal (availability of emotional support), belonging (availability of companions with whom one may engage in activities), and tangible support (availability of material aid) [58]. Items were rated on a four-point scale (definitely false, probably false, probably true, definitely true). Validation studies have indicated that it is possible to sum the items to create a global, first-order cumulative social support score [59], which was used herein. The total ISEL score could range from 12 to 48, with higher scores indicative of greater social support. Cronbach’s alpha for the ISEL in this sample was 0.80. 

Patient-provider communication was measured by four items from the Consumer Assessment of Healthcare Providers and Systems (CAHPS) survey [60]. Participants were asked how often in the past 12 months their doctor or health care provider: (1) listened carefully to them, (2) explained things in a way they could understand, (3) showed respect for what they had to say, and (4) spent enough time with them. Response options for each question were: never, sometimes, usually, and always. Cronbach’s alpha for these four items in this sample was 0.92. This measure’s scoring convention was based on whether each participant selected “always” for each item vs. did not select “always” for each item, essentially capturing ideal versus not always ideal patient-provider communication [61].

### 2.3. Data Analysis

The number of participants from the three churches totaled 1827 (*n* = 1325 Black women). For our study, only women ages 40 and older (*n* = 986) with complete data on the included measures (*n* = 832) comprised the sample analyzed. Differences between the included and excluded data were examined using t-tests and chi-square tests. 

The descriptive statistics of participants’ preferred and most recent sources of cancer information, mammography behavior, and covariates of interest were assessed. Participant characteristics relative to mammography behavior were examined using chi-square test or *t*-test for categorical and continuous variables, respectively.

Separate logistic regression analyses were conducted to measure associations between preferred source of cancer information and most recent source of cancer information, respectively, and mammography behavior. Both analyses controlled for recruitment site (i.e., at which of the three churches they were recruited) and the covariates described above. All analyses used two-tailed significance tests with a statistical significance level designated at *p* < 0.05. All analyses were conducted using SAS 9.4 [62]. 

## 3. Results

### 3.1. Patterns of Missingness

Compared to participants who had complete data on all the measures (*n* = 832), participants with missing data (*n* = 154) differed statistically on church recruitment site, health insurance status, annual household income level, cancer likelihood, and mammography behavior. Those with missing data were more likely than those with complete data to have been recruited from church site 3 (35.06% vs. 21.15%, *p* = 0.0009), were less likely to have health insurance (79.74% vs. 87.50%, *p* = 0.0103), were more likely to be from households with annual incomes between USD 40,000 and USD 79,999 (46.56% vs. 35.58%, *p* = 0.0482), considered themselves less likely to get cancer sometime in the future (very low: 46.88% vs. 36.42%, *p* = 0.0414), and were less likely to have received a mammogram in the last year (58.44% vs. 70.31%, *p* = 0.0036).

### 3.2. Participant Characteristics

Participants ranged from age 40 to 86 years (M = 54.80, SD = 8.89). Of the 832 participants, 70.31% (*n* = 585) had a mammogram in the last year. Overall, 11.18% of the sample (*n* = 93) reported achieving a high school degree or less, 39.18% attended some college (*n* = 326), and 49.64% reported acquiring at least a bachelor’s degree (*n* = 413). Additionally, 40.38% (*n* = 336) reported they were married/living with a partner, 87.50% (*n* = 728) were insured, 66.83% (*n* = 556) were employed, 38.82% (*n* = 323) reported ideal patient-provider communication, 2.40% (*n* = 20) had been diagnosed with at least 1 cancer, and 17.19% (*n* = 143) reported a family history of cancer(s) (Table 1). 

Compared with participants who did not receive a mammogram in the last year, those who did were older (55.87 vs. 52.25, *p* < 0.0001), and reported greater perceived social support (41.96 vs. 40.59, *p* = 0.0078). Chi-square tests showed significant associations between having received a mammogram in the last year and recruitment site (*p* = 0.0212), health insurance status (*p* < 0.0001), and patient-provider communication (*p* < 0.0001). Those reporting receiving a mammogram in the last year were more likely to have been recruited from church site 3 than were those who did not report receiving a mammogram (22.91% vs. 17.00%). They were also more likely to have had health insurance (92.82% vs. 74.90%) and have reported ideal patient-provider communication (44.62% vs. 25.10%) (Table 1).

### 3.3. The Associations between Preferred/Most Recent Source of Cancer Information and Mammography Behavior

There were no significant associations between “preferred source of cancer information” and mammography behavior (Table 2).

Results from adjusted logistic regression analyses revealed that women who most recently sought information from the internet had lower odds of having a mammogram in the last year than those who most recently sought information from a doctor or health provider (OR: 0.546, 95% CI: 0.336–0.886, *p* = 0.014). Compared with doctor or health provider, no significant difference in mammography behavior for any other “most recent source of information” options was found (Table 2). 

## 4. Discussion

### 4.1. Association between the Internet as an Information Source and Mammography Behavior

Over half of study participants (63.02%) most recently sought cancer information from the internet, and those who did so were less likely to have had a mammogram in the past year than those who sought information from a provider. The potential reasons for this association are plenty. Online health information is often unreliable and inaccessible due to lack of cultural sensitivity or appropriate reading level. Further, the tapestry of breast cancer screening guidelines available online can be confusing and conflicting. The prevalence of unreliable, inaccessible, and confusing sources of online cancer information may serve to enhance mammography inaction. Further, some sources may even directly endorse inaction (e.g., source recommending that Black women begin mammography at age 50 instead of 40).

Despite its flaws, the internet is likely to remain a prominent information source. Searching the internet for cancer information is cheaper, more convenient, and more private than scheduling an appointment with a provider. Some online sources, however, can take steps to attempt to mitigate the association between the internet as an information source and mammography inaction. Cancer organizations can update their websites to be written at a more globally accessible reading level. They can also ensure that those websites reflect Black faces and Black concerns. Ideally, the latter would include all of the organizations recommending that Black women begin having annual mammograms at age 40. At the very least, cancer organizations’ websites should provide information on the unique cancer risks faced by Black women, including a discussion of the benefits and harms of early mammography.

Black women should be informed that while the internet can be a valuable resource, the breast cancer information found there is not well-tailored to them and must therefore be read with a critical eye. Community organizations (including churches), cancer organizations (including those with an online presence), and even providers [63,64] should encourage individuals to use the internet in conjunction with, rather than in lieu of, conversations with providers. Providers can have conversations with patients about their online information-seeking tendencies [63] and, when applicable, point them toward reputable, accurate online information sources [64]. The quality of patient-provider communication has been shown in this study and others [65,66] to be positively associated with breast cancer screening uptake. Consequently, it may be that internet information-seeking can prompt thoughtful questions from patients, serving as a tool to potentially enhance communication with physicians and thereby increase mammography usage and reduce the breast cancer mortality disparity impacting Black women. 

### 4.2. Preferred Source of Cancer Information Was Not Predictive of Mammogram Usage

While the present study found that 55.41% of participants reported a doctor or health care provider as their preferred source of cancer information, there were no significant associations between any preferred source of cancer information and the likelihood of having had a mammogram in the last year. Preferred cancer information source appears to be unassociated with mammography behavior, but perhaps because it is scarcely associated with any behavior at all. It may merely be a preference that is not necessarily actualized. For example, while 55.41% of participants reported preferring to receive cancer information from a provider, only 17.88% actually turned to a provider when they most recently sought such information. Instead, most (63.02%) turned to the internet. The reasons for this may include that, as mentioned above, searching the internet for cancer information is cheaper, quicker, more convenient, and more private than scheduling an appointment with a provider.

Given the association between the internet as an information source and mammography inaction, intervention efforts should seek to ensure that Black women better actualize their reported preference for providers as their sources of cancer information. Health care providers are unlikely to successfully compete with the internet in terms of cost, timeliness, convenience, or privacy. Those designing interventions should therefore focus their efforts on educating Black women on the shortcoming of online information as well as its best uses, including as a supplement to conversations with providers. 

### 4.3. Likelihood of Mammogram Usage

Of the 832 Black women over age 40 included in the present sample, 70.31% reported having had a mammogram in the last year. This rate (from data collected in 2012) is slightly higher than but comparable to the rates observed by the Centers for Disease Control and Prevention (CDC) among Black women over 40 years of age nationwide in 2010 and 2015 (67.9 and 69.8, respectively) [67]. A CDC-observed rate for 2012 was not available. The mammography rate of 70.31% in the present study, however, fell short of the Healthy People 2020 target rate of 81.1% [9]. 

Black women face a unique combination of challenges to having mammograms. These include medical distrust born of past abuses and present experiences [68,69,70], less ideal patient-provider communication and its negative association with mammogram usage [52,71,72], and higher uninsured rates than their White counterparts [73]. The COVID-19 pandemic, along with the resulting guidelines discouraging nonurgent procedures, has exacerbated existing challenges to mammography and served as its own uptake challenge [74,75,76]. Therefore, a better understanding of additional factors that may affect mammography use among Black women, including preferred and actualized information sources about cancer, may be important to inform interventions to reach the Healthy People 2030 screening goal of 80.5% [10]. 

Increasing mammogram usage is particularly important in preserving the lives of Black women given their higher breast cancer mortality rates relative to White women [2]. Further observations from the present study provide insight into how interventions may be designed to accomplish such an increase. Compared to participants who did not receive a mammogram in the past year, those who did were significantly more likely to be older (55.87 vs. 52.25, *p* < 0.0001). This represents a potential opportunity to modify mammography behavior among Black women in favor of increased screening.

#### 4.3.1. Encourage Black Women to Begin Having Mammograms at Younger Ages

Despite the increased risk and potentially dire consequences, many Black women remain unaware of the breast cancer mortality disparity and how it impacts them. In one study conducted among Black women in Chicago, most participants believed that all women have an equal chance of dying from breast cancer [77]. Another study conducted among Black women in North Carolina found that a number of participants viewed breast cancer as a White disease, pointing out that breast cancer-related media rarely depict Black women [78]. These misconceptions underscore the importance of educating Black women about their unique breast cancer risk. This unique risk is precisely why there is growing support for Black women beginning mammography at age 40 [13,14,15,19,20,21,27]. Widespread effort should be made to educate Black women on their unique breast cancer risk. In fact, one recent study found that, regardless of age, race, or education, women prefer to be told about the benefits and harms of mammograms rather than simply being pushed to have them [79]. However, Black women should be encouraged not only to have mammograms but to do so at younger ages. Doing so would enable Black women to detect breast cancers at earlier, less malignant stages [5] when they are easier to treat and less fatal [6]. Stage at diagnosis does not account for all of the breast cancer mortality disparity [3], but studies suggest that early detection could reduce the disparity by 57% [27].

The breast cancer screening guidelines announced by prominent organizations, taken as a whole, may be more likely to confuse readers than to prompt mammogram appointments. They vary in terms of the frequency at which women should have mammograms and, importantly for the present discussion, the age at which women should begin them. None of the guidelines tailor their screening recommendations based on a woman’s race/ethnicity [25]. Given the known disparities in stage at diagnosis [3,4] and mortality [2], as well as recent evidence earlier screening may reduce those disparities [27], organizations promulgating breast cancer screening guidelines should strongly consider recommending that Black women begin mammography at age 40.

#### 4.3.2. Covariates

Consistent with other studies in the literature, several covariates contributed unique variance to likelihood of having had a mammogram in the last year. Specifically, compared to participants who did not receive a mammogram in the past year, those who did were significantly more likely to report greater perceived social support (41.96 vs. 40.59, *p* = 0.0078), carry health insurance (*p* < 0.0001), and report better patient-provider communication (*p* < 0.0001). Previous studies have found similar results with respect to insurance status among Black church-going women aged 40 and older [52] and among Black women generally [80]. One study found similar results with respect to patient-provider communication among Black church-going women aged 40 and older [52]. Other studies have found similar results with respect to perceived social support, but among older Black women [81,82] or women of all races aged 40 and older [83]. The results of the present study support or extend these findings’ applicability to Black church-going women aged 40 and older. Implications of these findings include that mammography uptake may also be increased among this group by (1) increasing their actual and/or perceived social support (e.g., hosting community events that enable women to network with others to form social bonds or that affirm women and meet their concerns with spiritual comfort, making women aware of such existing events, or connecting women with individuals and organizations that offer tangible support such as assistance with chores [84,85,86,87,88]), (2) mitigating insurance coverage as a barrier to their mammogram usage (e.g., supporting measures to improve the affordability of health insurance, informing Black women of low-cost health insurance options, arranging for mobile mammogram units in the community [89]), and/or (3) improving the patient-provider communication they experience (e.g., training providers to communicate in ways that ware more patient-centered and less verbally dominant [90], teaching patients to ask providers clear and direct questions to obtain the information they need to make health care decisions, or encouraging patients to consider changing providers if communication with their current provider is nonideal and irreparable).

#### 4.3.3. Role of the Church

The Black Church can educate Black women about their unique breast cancer risk, the benefits and harms of mammography, and evidence showing the values of earlier mammogram usage for Black women. This could take the form of messages from the pulpit, messages in church newsletters or on church websites, or any of a number of other forms of health ministry. In fact, most Black adults say that it is essential for churches to offer a sense of community or fellowship and to offer spiritual comfort (71% and 72%, respectively) [91]. Research has shown that church-based mammogram education is positively associated with mammogram uptake and that the association is strengthened by receiving mammogram education numerous times at church [52]. Accordingly, churches should provide this information as often as possible and in as many outlets as possible.

The Church can also serve as a social network and encourage its members to support one another. It can host social events and partner with experts to educate its congregants on the breast cancer screening benefits available through Medicaid, Medicare, or any health insurance plans they may carry. Where feasible, churches can also partner with health care providers and community organizations to provide free mammograms. The Church can host coaching sessions whereby providers train congregants on how to ask questions of their providers and/or how to identify a new provider. Whether it be through direct education or any number of partnerships, the present study identifies several factors that the Black Church is well-positioned to address should it seek to increase mammography among Black women.

### 4.4. Limitations

Data analyzed in this study were cross-sectional, relied on self-reported mammogram behavior, and were collected in 2012. Further research is necessary to confirm the persistence of these outcomes in light of the COVID-19 pandemic and changes in internet usage over the past decade. More people access the internet today [56], and that access is more often unfettered due to increased ownership of smartphones [92,93]. Greater access to the internet may equate with increased use of it as a cancer information seeking tool. Indeed, in an October 2018 national survey of 164 Black women, Francis and Zeyala found that 77% of those who sought cancer information did so online [94]. Additionally, individuals accessing the internet are more likely than they were ten years ago to spend their time on social media [95,96]. Despite being known to facilitate the spread of misinformation [97] and disinformation [98], social media platforms have become a prominent source of health information over the past ten years [99] and have been shown to influence health behavior [100]. Yet, recent data continue to suggest that the available cancer information may be particularly problematic for Black consumers. For example, in a March 2022 publication assessing representation in online prostate cancer content, Loeb et al. reviewed 81 websites and 127 videos [101]. They found the same issues that have been reviewed within this study about online breast cancer content [22,24,25,32,33,34,35,36,37,38,40]; most lacked any perceived Black representation and did not discuss racial/ethnic disparities [101]. Of the content that did have Black representation, none was found to be high quality and understandable [101]. In a 2018 study that included 50 in-depth interviews with Black and White women, Padamsee et al. concluded that Black women were less aware of their breast cancer prevention options due to structural, social, and interpersonal barriers in accessing such information [102]. Additionally, in a series of focus groups conducted in December 2020 and January 2021 with 33 women (28 identifying ad Black, 4 as Afro-Caribbean, and 1 as Afro-Latina) in New York and New Jersey, Bea et al. found that several participants expressed skepticism or misunderstanding of mammograms, including the belief that the mammogram itself puts a patient at risk of getting breast cancer [103]. Thus, although additional studies with more recent data would be helpful to better understand changes in associations between the internet/social media as a cancer information source and mammography uptake over the last decade, the context of increased internet use coupled with a persistent lack of representation or race-tailored screening guidelines for Black women may suggest the continuing relevance of the current findings. Further, the Black Church, in particular, may continue to play a crucial role in implementing interventions tailored to Black women given the ubiquity of misinformation and disinformation spread via social media. 

Of the sample analyzed in the present study, 49.64% reported having acquired at least a bachelor’s degree. This is notably higher than the 36.1% of Black women estimated to hold college degrees nationwide [104]. The participants in the sample may be better able to understand breast cancer screening recommendations and navigate the healthcare system than are Black women nationwide and may therefore be uncharacteristically likely to receive mammograms. Future studies should aim to have an analytic sample with educational backgrounds reflective of Black women nationwide for increased generalizability. The analyzed sample was composed entirely of Black, church-going women in Houston, Texas. This may affect generalizability to Black women nationwide who may or may not attend church services. 

As noted in Section 3.1 above, 154 respondents with missing data were excluded from the present analyses. Compared to the 832 participants who had all data and were included, the excluded respondents were more likely to have been recruited from church site 3, less likely to have health insurance, and more likely to be from households with annual incomes between USD 40,000 and USD 79,999. The excluded respondents also considered themselves less likely to get cancer sometime in the future and were less likely to have received a mammogram in the last year. Future studies should aim to have broader socioeconomic representation among the analytic sample so that stronger inferences can be drawn with respect to the general population.

## 5. Conclusions

This study adds to the extant literature by evidencing an association between the internet as a cancer information source and mammography behavior among Black church-going women. The present study finds that those who most recently sought cancer information from the internet are less likely to have had a mammogram in the last year than are those who most recently sought such information from a provider. The study also expands upon existing literature by supporting previously found associations between, on one hand, carrying health insurance and/or ideal patient-provider communication and, on the other hand, a greater likelihood of recent mammography. Further, the study finds older age and greater perceived social support to be positively correlated with recent mammography usage. These results have many implications for the design of future interventions aimed at increasing mammogram usage among Black women aged 40 and older. Black women should be widely and frequently educated about their unique breast cancer risks, including by organizations promulgating mammography guidelines. New avenues of social support should be made available to Black women, and existing avenues should be brought to their attention. Efforts should be made to increase knowledge of mammogram coverage under Medicaid, Medicare, and existing insurance plans, as well as to bring free mammogram services to women who are underinsured. Both patients and providers should endeavor to improve patient-provider communication. Online cancer information sources should work to improve their reliability, cultural sensitivity, and readability, making sure to highlight the unique risks facing Black women. Cancer organizations and providers should work to ensure that the internet serves as a patient resource rather than an impediment to mammogram usage. The Black Church is well-positioned to lead or assist in all of the aforementioned efforts. 

Internet usage and content have expanded with the proliferation of smartphones and social media accounts in the years since the data analyzed in this study were collected, but prominent online breast cancer information sources continue to lack representation and race-tailored screening recommendations for Black women. Accordingly, further research is necessary to confirm the persistence of the associations discussed herein.

## Figures and Tables

**Table 1:** Participant Characteristics Relative to Having Received a Mammogram Within the Last Year (N = 832 Black, church-going women over age 40).

	All Participants	Did Not Receive Mammogram	Received Mammogram	Statistics	*p*-Value
	(*n* = 832)	(*n* = 247)	(*n* = 585)		
	Mean (SD)/% [*n*]				
Age	54.80 (8.89)	52.25 (8.46)	55.87 (8.86)	2.82	<0.0001 ***
Education				3.11	0.2115
≤High school	11.18 [93]	12.15 [30]	10.77 [63]		
Some college	39.18 [326]	42.91 [106]	37.61 [220]		
≥Bachelor’s degree	49.64 [413]	44.94 [111]	51.62 [302]		
Partner status				0.79	0.3739
Other *	59.62 [496]	61.94 [153]	58.63 [343]		
Married/living with a partner	40.38 [336]	38.06 [94]	41.37 [242]		
Church Site				7.70	0.0212 **
Church 1	11.54 [96]	63.56 [157]	64.10 [375]		
Church 2	10.22 [85]	19.43 [48]	12.99 [76]		
Church 3	0.96 [8]	17.00 [42]	22.91 [134]		
Health Insurance Coverage				51.00	<0.0001 ***
No	12.50 [104]	25.10 [62]	7.18 [42]		
Yes	87.50 [728]	74.90 [185]	92.82 [543]		
Annual Household Income				2.71	0.2577
<USD 40,000	31.13 [259]	34.41 [85]	29.74 [174]		
USD 40,000–USD 79,999	35.58 [296]	36.03 [89]	35.38 [207]		
≥USD 80,000	33.29 [277]	29.55 [73]	34.87 [204]		
Employment Status				0.02	0.8799
Unemployed	33.17 [276]	32.79 [81]	33.33 [195]		
Employed	66.83 [556]	67.21 [166]	66.67 [390]		
Patient-Provider communication				27.85	<0.0001 ***
Not always ideal	61.18 [509]	74.90 [185]	55.38 [324]		
Ideal	38.82 [323]	25.10 [62]	44.62 [261]		
Social support	41.55 (6.46)	40.59 (6.96)	41.96 (6.20)	2.67	0.0078 **
Personal diagnosis of cancer				2.12	0.1456
No	97.60 [812]	98.79 [244]	97.09 [568]		
Yes	2.40 [20]	1.21 [3]	2.91 [17]		
Family diagnosis of cancer				0.80	0.3704
No	82.81 [689]	84.62 [209]	82.05 [480]		
Yes	17.19 [143]	15.38 [38]	17.95 [105]		
Cancer likelihood				3.06	0.5479
Very low	36.42 [303]	38.87 [96]	35.38 [207]		
Somewhat low	25.36 [211]	21.46 [53]	27.01 [158]		
Moderate	30.41 [253]	31.98 [79]	29.74 [174]		
Somewhat high	6.85 [57]	6.88 [17]	6.84 [40]		
Very high	0.96 [8]	0.81 [2]	1.03 [6]		
Cancer relative risk				1.11	0.5747
More likely to get cancer	8.05 [67]	7.29 [18]	8.38 [49]		
Less likely	56.37 [469]	59.11 [146]	55.21 [323]		
About as likely	35.58 [296]	33.60 [83]	36.41 [213]		
Cancer worries				0.98	0.9131
Never	26.44 [220]	27.13 [67]	26.15 [153]		
Rarely	37.62 [313]	37.25 [92]	37.78 [221]		
Sometimes	29.21 [243]	29.96 [74]	28.89 [169]		
Often	4.93 [41]	4.45 [11]	5.13 [30]		
All the time	1.80 [15]	1.21 [3]	2.05 [12]		
Preferred source of cancer information				7.02	0.1351
Other media	11.54 [96]	14.17 [35]	10.43 [61]		
Cancer organization	10.22 [85]	11.74 [29]	9.57 [56]		
Social network	0.96 [8]	1.62 [4]	0.68 [4]		
Doctor or health care provider	55.41 [461]	49.39 [122]	57.95 [339]		
Internet	21.88 [182]	23.08 [57]	21.37 [125]		
Most recent source of cancer information				6.68	0.1539
Other media	12.04 [99]	12.76 [31]	11.74 [68]		
Cancer organization	4.50 [37]	4.53 [11]	4.49 [26]		
Social network	2.55 [21]	3.29 [8]	2.25 [13]		
Doctor or health care provider	17.88 [147]	12.76 [31]	20.03 [116]		
Internet	63.02 [518]	66.67 [162]	61.49 [356]		

Note. Social support was measured with the Interpersonal Support Evaluation List. Patient-provider communication was measured with the Consumer Assessment of Healthcare Providers and Systems. Cancer likelihood was how likely the participant thought it was that they would develop cancer in the future. Cancer relative risk was self-assessed personal likelihood of getting cancer compared to the average person. Cancer worries was how often participants worried about getting some type of cancer. SD = Standard deviation. * = Other included divorced, widowed, separated, and never married. ** = *p* <0.05. *** = *p* <0.0001.

**Table 2:** Logistic Regression Analyses Examining Associations Between Preferred and Most Recent Source of Cancer Information, Respectively, and Having Received a Mammography in the Last Year (N = 832 Black, church-going women).

Effect	Estimate	SE	OR	95% CI	*p*-Value
Preferred Source of Cancer Information					
Cancer organization (ref: doctor/health care provider)	−0.391	0.269	0.677	(0.399, 1.147)	0.147
Internet (ref: doctor/health care provider)	−0.155	0.203	0.857	(0.575, 1.276)	0.447
Other media (ref: doctor/health care provider)	−0.415	0.260	0.660	(0.397, 1.099)	0.111
Social network (ref: doctor/health care provider)	−0.533	0.805	0.587	(0.121, 2.840)	0.508
Church site 2 (ref: Church site 1)	−0.501	0.235	0.606	(0.382, 0.960)	0.033 **
Church site 3 (ref: Church site 1)	0.148	0.219	1.159	(0.755, 1.780)	0.500
Age	0.018	0.008	1.018	(1.001, 1.034)	0.033 **
Education (≥Bachelor’s degree) (ref: ≤High school)	0.138	0.297	1.148	(0.641, 2.056)	0.642
Education (Some college) (ref: ≤High school)	−0.163	0.280	0.850	(0.491, 1.471)	0.561
Partner status (Married/Living with a partner) (ref: Other *)	0.145	0.186	1.156	(0.803, 1.665)	0.435
Health insurance coverage (ref: No)	1.109	0.247	3.031	(1.868, 4.916)	<0.001 ***
Annual household income (USD 40,000–USD 79,999) (ref: <USD 40,000)	−0.183	0.224	0.833	(0.537, 1.292)	0.415
Annual household income (≥USD 80,000) (ref: <USD 40,000)	−0.212	0.266	0.809	(0.480, 1.364)	0.427
Employment status (ref: Unemployed)	0.045	0.195	1.046	(0.714, 1.533)	0.816
Patient-provider communication (ref: Not Always Ideal)	0.783	0.183	2.187	(1.527, 3.133)	<0.001 ***
Social support	−0.009	0.011	0.991	(0.969, 1.013)	0.412
Personal diagnosis of cancer (ref: No)	0.661	0.676	1.936	(0.515, 7.278)	0.328
Family member diagnosed with cancer (ref: No)	0.268	0.231	1.307	(0.831, 2.057)	0.247
Cancer likelihood (Moderate) (ref: Very low)	−0.352	0.239	0.704	(0.441, 1.123)	0.141
Cancer likelihood (Somewhat high) (ref: Very low)	−0.597	0.397	0.550	(0.253, 1.199)	0.133
Cancer likelihood (Somewhat low) (ref: Very low)	0.109	0.226	1.115	(0.716, 1.739)	0.629
Cancer likelihood (Very high) (ref: Very low)	−0.541	1.019	0.582	(0.079, 4.287)	0.595
Cancer relative risk (About as likely) (ref: More likely)	−0.492	0.360	0.611	(0.302, 1.237)	0.171
Cancer relative risk (Less likely) (ref: More likely to get cancer)	−0.778	0.374	0.459	(0.221, 0.955)	0.037 **
Cancer worries (All the time) (ref: Never)	0.916	0.763	2.500	(0.561, 11.151)	0.230
Cancer worries (Often) (ref: Never)	0.162	0.433	1.175	(0.503, 2.747)	0.709
Cancer worries (Rarely) (ref: Never)	0.012	0.218	1.012	(0.660, 1.553)	0.955
Cancer worries (Sometimes) (ref: Never)	−0.041	0.244	0.960	(0.595, 1.549)	0.868
Most Recent Source of Cancer Information					
Cancer organization (ref: doctor/health care provider)	−0.686	0.441	0.504	(0.212, 1.195)	0.120
Internet (ref: doctor/health care provider)	−0.605	0.247	0.546	(0.336, 0.886)	0.014 **
Other media (ref: doctor/health care provider)	−0.505	0.322	0.604	(0.321, 1.134)	0.117
Social network (ref: doctor/health care provider)	−0.883	0.515	0.414	(0.151, 1.136)	0.087
Church site 2 (ref: Church site 1)	−0.509	0.238	0.601	(0.377, 0.957)	0.032 **
Church site 3 (ref: Church site 1)	0.183	0.221	1.200	(0.778, 1.851)	0.409
Age	0.018	0.008	1.018	(1.001, 1.035)	0.036 **
Education (≥Bachelor’s degree) (ref: ≤High school)	0.204	0.311	1.227	(0.667, 2.255)	0.511
Education (Some college) (ref: ≤High school)	−0.079	0.293	0.924	(0.520, 1.640)	0.787
Partner status (Married/Living with a partner) (ref: Other *)	0.123	0.187	1.131	(0.783, 1.633)	0.511
Health insurance coverage (ref: No)	1.144	0.246	3.140	(1.939, 5.085)	<0.001 ***
Annual household income (USD 40,000–USD 79,999) (ref: <USD 40,000)	−0.124	0.226	0.884	(0.568, 1.375)	0.584
Annual household income (≥USD 80,000) (ref: <USD 40,000)	−0.126	0.270	0.882	(0.519, 1.497)	0.641
Employment status (ref: Unemployed)	0.098	0.196	1.102	(0.751, 1.618)	0.618
Patient-provider communication (ref: Not Always Ideal)	0.750	0.185	2.116	(1.472, 3.042)	<0.001 ***
Social Support	−0.006	0.012	0.994	(0.971, 1.016)	0.576
Personal diagnosis of cancer (ref: No)	0.395	0.675	1.484	(0.395, 5.575)	0.559
Family member diagnosed with cancer (ref: No)	0.242	0.231	1.274	(0.810, 2.003)	0.296
Cancer likelihood (Moderate) (ref: Very low)	−0.400	0.241	0.670	(0.418, 1.074)	0.096
Cancer likelihood (Somewhat high) (ref: Very low)	−0.648	0.401	0.523	(0.238, 1.147)	0.106
Cancer likelihood (Somewhat low) (ref: Very low)	0.082	0.228	1.086	(0.694, 1.697)	0.719
Cancer likelihood (Very high) (ref: Very low)	−0.494	1.025	0.61	(0.082, 4.546)	0.630
Cancer relative risk (About as likely) (ref: More likely)	−0.370	0.359	0.691	(0.342, 1.396)	0.303
Cancer relative risk (Less likely) (ref: More likely to get cancer)	−0.687	0.374	0.503	(0.242, 1.048)	0.066
Cancer worries (All the time) (ref: Never)	1.026	0.775	2.791	(0.611, 12.755)	0.186
Cancer worries (Often) (ref: Never)	0.307	0.450	1.359	(0.562, 3.284)	0.495
Cancer worries (Rarely) (ref: Never)	0.058	0.221	1.060	(0.687, 1.636)	0.792
Cancer worries (Sometimes) (ref: Never)	−0.013	0.247	0.987	(0.608, 1.602)	0.958

Note. Social support was measured with the Interpersonal Support Evaluation List. Patient-provider communication was measured with the Consumer Assessment of Healthcare Providers and Systems. Cancer likelihood was how likely the participant thought it was that they would develop cancer in the future. Cancer relative risk was self-assessed personal likelihood of getting cancer compared to the average person. Cancer worries was how often participants worried about getting some type of cancer. SD = Standard deviation. SE = Standard Error; OR = Odds Ratio; CI = Confidence Interval; * = Other included divorced, widowed, separated, and never married; ** = *p* < 0.05; *** = *p* < 0.001.

## Data Availability

The data are not publicly available due to ethical restrictions based on informed consent agreements that did not specify broad data release. Additionally, we worked with the churches’ leadership directly for permission to conduct the broader study, which banked sensitive data (e.g., buccal saliva sample); the broad release of de-identified data was not something that the churches agreed to for privacy/confidentiality reasons. Data may be available from the Ancillary Studies Committee (PI: McNeill) at the University of Texas MD Anderson Cancer Center for researchers who meet the criteria for access to confidential data. Interested researchers may contact Office of Human Subjects Protection at MD Anderson Cancer Center at IRB_Help@mdanderson.org or at 713-792-6477.
